# Concentration of antibodies against *Porphyromonas gingivalis* is increased before the onset of symptoms of rheumatoid arthritis

**DOI:** 10.1186/s13075-016-1100-4

**Published:** 2016-09-07

**Authors:** Linda Johansson, Natalia Sherina, Nastya Kharlamova, Barbara Potempa, Barbro Larsson, Lena Israelsson, Jan Potempa, Solbritt Rantapää-Dahlqvist, Karin Lundberg

**Affiliations:** 1 Public Health and Clinical Medicine/Rheumatology, Umeå University, Umeå, Sweden; 2 Rheumatology Unit, Department of Medicine Solna, Karolinska Institutet, Stockholm, Sweden; 3 Department of Oral Immunology and Infectious Diseases, University of Louisville, School of Dentistry, Louisville, KY USA; 4 Department of Microbiology, and Malopolska Centre of Biotechnology, Faculty of Biochemistry, Biophysics and Biotechnology, Jagiellonian University, Krakow, Poland

**Keywords:** *Porphyromonas gingivalis*, Antibodies, Arginine gingipainB, Citrullinated peptide, Rheumatoid arthritis, Predating onset, Anti-citrullinated protein/peptide antibodies

## Abstract

**Background:**

The periodontal pathogen *Porphyromonas gingivalis* is hypothesized to be important in rheumatoid arthritis (RA) aetiology by inducing production of anti-citrullinated protein antibodies (ACPA). We have shown that ACPA precede RA onset by years, and that anti-*P. gingivalis* antibody levels are elevated in RA patients. The aim of this study was to investigate whether anti-*P. gingivalis* antibodies pre-date symptom onset and ACPA production.

**Methods:**

A case–control study (251 cases, 198 controls) was performed within the Biobank of Northern Sweden. Cases had donated blood samples (*n* = 422) before the onset of RA symptoms by 5.2 (6.2) years (median (interquartile range)). Blood was also collected from 192 RA patients following diagnosis. Antibodies against *P. gingivalis* virulence factor arginine gingipainB (RgpB), and a citrullinated peptide (CPP3) derived from the *P. gingivalis* peptidylarginine deiminase enzyme, were analysed by ELISA.

**Results:**

Anti-RgpB IgG levels were significantly increased in pre-symptomatic individuals (mean ± SEM; 152.7 ± 14.8 AU/ml) and in RA patients (114.4 ± 16.9 AU/ml), compared with controls (*p* < 0.001). Anti-CPP3 antibodies were detected in 5 % of pre-symptomatic individuals and in 8 % of RA patients, with elevated levels in both subsets (4.33 ± 0.59 and 9.29 ± 1.81 AU/ml, respectively) compared with controls (*p* < 0.001). Anti-CPP3 antibodies followed the ACPA response, with increasing concentrations over time, whilst anti-RgpB antibodies were elevated and stable in the pre-symptomatic individuals with a trend towards lower levels after RA diagnosis.

**Conclusions:**

Anti-*P. gingivalis* antibody concentrations were significantly increased in RA patients compared with controls, and were detectable years before onset of symptoms of RA, supporting an aetiological role for *P. gingivalis* in the development of RA.

**Electronic supplementary material:**

The online version of this article (doi:10.1186/s13075-016-1100-4) contains supplementary material, which is available to authorized users.

## Background

Rheumatoid arthritis (RA), a complex chronic inflammatory disease with a worldwide prevalence of 0.5–1.0 % [1], is characterized by production of anti-citrullinated protein/peptide antibodies (ACPA) in the majority of patients and persistent inflammation in the synovial tissue of the joints leading to destruction of cartilage and bone [2–4]. The aetiology of RA remains unknown, although a complex interplay exists between genetic and environmental factors [5, 6]. Chronic periodontitis (PD), which is the world’s commonest inflammatory disease often resulting in destruction of alveolar bone and tooth loss, has been suggested as an environmental determinant for the occurrence and severity of RA [7–9].

Elucidation of the potential aetiological link between PD and RA has progressed [8, 10, 11] and a number of studies have identified similarities between these two diseases that possibly explain the epidemiological association. Both PD and RA display systemic markers of inflammation (e.g. C-reactive protein and pro-inflammatory cytokines) [6, 12, 13], and an association with *HLA-DRB1* alleles [14] and smoking [15, 16] has been described for both RA and PD. Furthermore, citrullinated proteins have been detected in both rheumatoid joints and inflamed gingival tissue, as well as in other tissues in relation to inflammatory conditions [17–19]. However, an association between PD and established RA could not be confirmed in one of our recent publications [20].

Moreover, data from one of our other studies suggest that the oral pathogen *Porphyromonas gingivalis*, rather than PD, may be linked to the development of RA [21]. *P. gingivalis* is a common periodontal pathogen associated with PD [22, 23], and is the only prokaryote known to express an endogenous peptidylarginine deiminase enzyme (*P*.PAD), a virulence factor capable of citrullinating human and bacterial proteins, including auto-citrullination [24–26]. *P.*PAD interacts with another major virulence factor, arginine gingipainB (RgpB), an arginine-specific extra-cellular protease expressed on the surface of the bacterial outer membrane [24, 25]. RgpB is essential for *P. gingivalis* to citrullinate peptides; that is, only after degradation by RgpB can *P.*PAD convert peptidylarginine into peptidylcitrulline [24, 26].

It is hypothesized that citrullination by *P. gingivalis* causes a chronic exposure of citrullinated peptides in the inflamed periodontium, possibly leading to a break of immune tolerance in genetically susceptible individuals and subsequent production of ACPA. Epitope spreading, induced by molecular mimicry and cross-reactivity with citrullinated epitopes exposed in the joint, could lead to progression of chronic RA [27–29]. ACPA appear many years before the onset of RA [30, 31], suggesting that the initial immune dysregulation occurs long before symptoms of RA develop, outside the joints, potentially at mucosal sites such as the gingival tissue [17, 27, 32]. Citrullinated *P.*PAD has been demonstrated to be a target of the ACPA response [25] and we recently demonstrated that elevated anti-RgpB antibody levels have a stronger association with RA than smoking [21], identifying *P. gingivalis* as a potential mechanistic link between PD and RA. Importantly, in the same study we could also show that anti-RgpB IgG levels were significantly increased in sera from PD patients compared with periodontally healthy individuals, supporting anti-RgpB IgG as a surrogate marker for oral infection by *P. gingivalis.*

This study investigated whether raised anti-*P. gingivalis* antibody levels precede onset of symptoms of RA and the ACPA response in order to elucidate the role of *P. gingivalis* as a potential trigger of autoimmunity and the development of RA. Importantly, because we have no data on periodontal status in our cohorts, we have only focused on *P. gingivalis*, not on PD, in the present study. Consequently, we analysed the antibody response to RgpB and CPP3, a citrullinated peptide derived from *P.*PAD, in blood samples collected prior to the onset of symptoms and at the diagnosis of RA. Identifying a potential mechanism capable of breaking immunological tolerance at the earliest stage of disease would provide an insight into the aetiopathology of RA, and could indicate new clinical therapies and interventions.

## Methods

### Study population

A case–control study was performed within the Medical Biobank of Northern Sweden and the Maternity cohort. The cohorts within the Medical Biobank are population-based health surveys, and all habitants of Västerbotten county are continuously invited to participate. Information concerning recruitment, blood sampling and storage conditions has been described previously [30]. The Maternity cohort is based on blood samples collected from pregnant women being screened for immunity to rubella [30]. To identify individuals having donated blood samples prior to onset of symptoms of RA, the register of patients fulfilling the 1987 classification criteria for RA [33] was linked to those of the Medical Biobank and the Maternity cohort.

The study included 251 pre-symptomatic individuals (58 men/193 women, mean age ± SD 50.5 ± 11.9 years) who had donated a total of 422 plasma/serum samples (375 from the Biobank cohorts and 47 from the Maternity cohort) at different time points pre-dating onset of RA symptoms. From the same cohorts, 198 (31 men/167 women, mean age ± SD 49.3 ± 14.8 years) population-based controls (173 from the Biobank cohorts and 25 from the Maternity cohort), matched for sex and age, with sufficient plasma/serum volumes, were identified. The median time pre-dating the onset of RA symptoms was 5.2 years (interquartile range 6.2). At least one sample was identified for each of the 251 pre-symptomatic individuals; two samples were identified for 92 individuals (36.6 %), three samples for 46 individuals (18.3 %), four samples for 22 individuals (8.8 %), five samples for nine individuals (3.6 %) and six samples for two individuals (0.8 %). At the time of RA diagnosis (≤12 months of symptoms), 192 patients (144 females/48 males) donated blood samples at the early arthritis clinic. One hundred and fifty-three of these early RA patients were identified within the group of pre-symptomatic individuals because they had donated blood samples before onset of symptoms (median 5.6 years, IQR 6.3). Data detailing periodontal status or treatment were not available for these cases. The participants gave their written informed consent and the Regional Ethics Committee at Umeå University approved the study.

Smoking status was defined as ever smoker (including former and current smokers), current smoker or never smoker. Because information on being either former or current smoker was lacking for a number of cases, we present data for both groups. Of the pre-symptomatic individuals, 64 % (160/250) were ever smokers and 25.6 % (55/215) were current smokers, while 49.2 % (89/181) of the controls were ever smokers and 13.1 % (18/137) were current smokers. *HLA-DRB1* shared epitope (SE) alleles (0101/0401/0404/0405/0408) and *PTPN22* 1858C/T polymorphism were genotyped as described previously [34, 35]. Demographic data for the three groups are presented in Table 1.

**Table 1:** Descriptive data of the pre-symptomatic individuals, patients with RA and controls

	Pre-symptomatic individuals^a^ (*n* = 251)	RA patients (*n* = 192)	Controls (*n* = 198)
Female sex, *n* (%)	193 (76.9)	144 (75.0)	167 (84.3)
Age, mean ± SD years	50.5 (11.9)^b^	56.5 (11.3)	49.3 (14.8)
Ever smoker, *n* (%)	160/250 (64.0)	129 (67.2)	89/181 (49.2)
*HLA-DRB1* SE positive^c^, *n* (%)	166/250 (66.4)	122/192 (63.5)	61/171 (35.7)
*PTPN22* 1858 T carrier, *n* (%)	86/250 (34.4)	63 (32.8)	32/173 (18.5)

^a^Pre-symptomatic individuals were defined as individuals before the onset of symptom of RA

^b^Mean age, as calculated for all samples when collected, pre-symptomatic individuals: *n* = 422

^c^ *HLA*-*DRB1* shared epitope (*SE*) alleles were defined as 0101/0401/0404/0405/0408

*RA* rheumatoid arthritis, *SD* standard deviation

### Antibody analysis

Using in-house ELISAs as described previously [21, 25], all plasma/serum samples were analysed blinded for antibodies against the RgpB protein purified from *P. gingivalis* [36], a synthetic cyclic citrullinated peptide (CPP3) derived from *P.*PAD and the corresponding arginine-containing control peptide (RPP3) (Innovagen AB, Lund, Sweden). Serial dilutions of antibody-positive serum pools (anti-RgpB and anti-CPP3 IgG, respectively) were included on all ELISA plates to generate standard curves in order to compare antibody concentrations between cases analysed on different ELISA plates. All antibody levels are presented as arbitrary units/ml (AU/ml).

Treating anti-CPP3 IgG as a “classical” ACPA, a cut-off value for antibody positivity was defined using receiver operating characteristic (ROC) curves, based on the anti-CPP3 IgG responses in RA patients and controls. The cut-off value for positivity was set at >29.19 AU/ml, giving a specificity of 96 %. No cut-off value could be assigned for the anti-RgpB IgG response, due to a lack of data regarding PD status of the study subjects.

The plasma/serum samples included in this study had been analysed previously for the presence of antibodies against cyclic citrullinated peptides (CCPs), using the anti-CCP2 ELISA assay (Euro Diagnostica, Malmö, Sweden), and different ACPA fine specificities (e.g. cfibrinogenβ36-52 (Fibβ36-52, citrullinated at position 44), α-enolase5-21 (CEP-1, citrullinated at position 9 and 15) and cfilaggrin307-324 (CCP1, citrullinated at position 13)) using a microarray based on the ImmunoCAP ISAC system (PhaDia AB, Uppsala, Sweden) [37]. Analysis of rheumatoid factor (RF) of IgM isotype was performed using the EliA assay on the Phadia 2500-system according to the manufacturer’s instructions (Phadia GmbH, Freiburg, Germany). The cut-off value with the optimum sensitivity and specificity was that with 96 % specificity.

### Statistical analysis

Continuous data were compared using a non-parametric Mann–Whitney U-test/Wilcoxon signed rank test including two groups, and a Kruskal–Wallis test including several groups. The chi-square test or Fisher’s exact test was used when analysing categorical data. Correlation analysis was performed using Spearman’s rank correlation. Because of the lack of a cut-off value for the anti-RgpB antibody response, we stratified the anti-RgpB antibody concentrations into above or below the 75th percentile in the analyses. Logistic regression analysis was used to identify associations between antibodies and risk factors in the development of RA adjusted for age and sex. Associations were presented as odds ratios (ORs) with 95 % confidence interval (CI). Standard methods were used for analysing interactions [38]. All adjustments were based on previously performed studies and hypothesis. The statistical analyses were performed using SPSS 23.0 software (Chicago, IL, USA).

## Results

### The anti-RgpB antibody response and anti-CPP3 antibody response in pre-symptomatic individuals, RA patients and controls

**Fig. 1:**
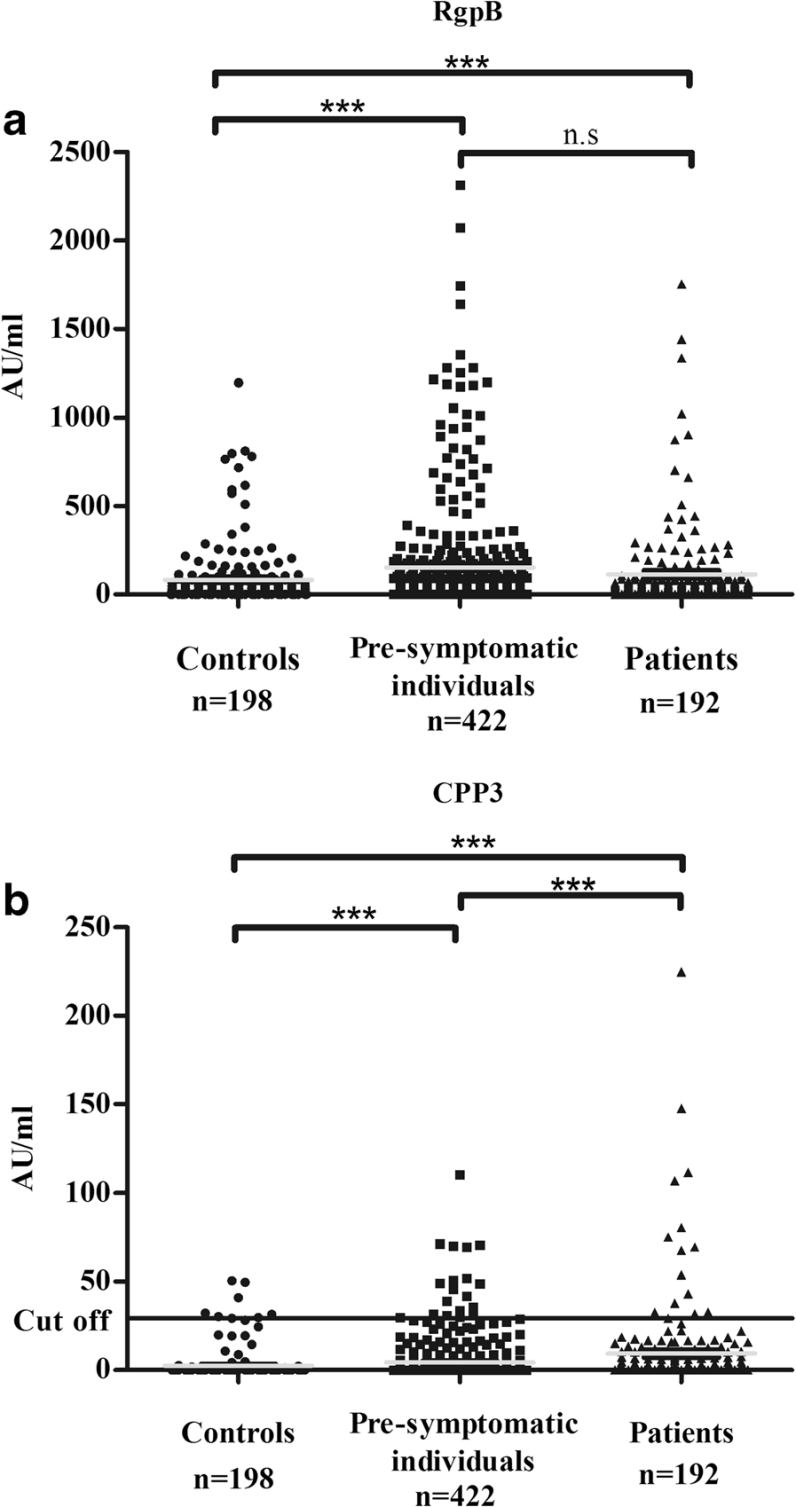
Concentrations of anti-RgpB (**a**) and anti-CPP3 (**b**) antibodies in controls, pre-symptomatic individuals and patients with RA. Mean concentrations marked as a *grey line*. ***p < 0.001. *n.s* not significant

The concentration of anti-RgpB IgG was significantly increased in RA patients (mean ± SEM 114.4 ± 16.9 AU/ml) and in particular in pre-symptomatic individuals, calculated using all 422 samples (152.7 ± 14.8 AU/ml) or one sample per individual (when more than one sample was available, the sample closest to symptom onset was chosen) (133.4 ± 16.2 AU/ml; data not shown), compared with control subjects (82.2 ± 12.1 AU/ml, *p* < 0.001 for all three groups) (Fig. 1a).

The anti-CPP3 IgG levels were significantly increased in RA patients (mean ± SEM 9.29 ± 1.81 AU/ml) compared with pre-symptomatic individuals, both when all samples were analysed (4.33 ± 0.59 AU/ml) (Fig. 1b) and when only the sample closest to disease onset was analysed (5.56 ± 0.89 AU/ml; data not shown); both comparisons were analysed at group level (*p* < 0.001). Antibody concentrations in both RA patients and pre-symptomatic individuals were also significantly increased compared with controls (2.36 ± 0.58 AU/ml, *p* < 0.001) (Fig. 1b).

The frequency of anti-CPP3 antibodies was 4.5 % in pre-symptomatic individuals when calculated for all 422 samples, or 6.8 % when calculated for the 251 individuals who were ever positive, and 7.8 % in RA patients (data not shown). Less than 2 % of all individuals showed reactivity towards the arginine-containing control peptide RPP3 (data not shown).

**Fig. 2:**
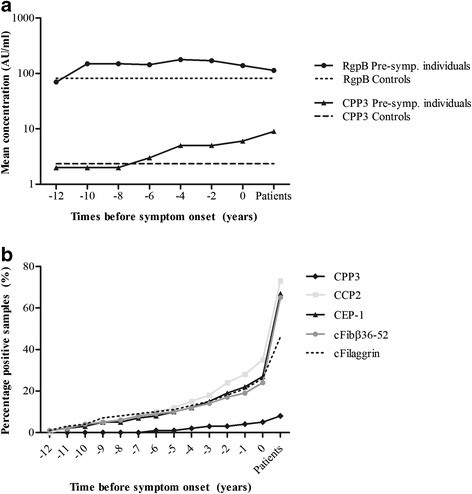
Antibody responses during the pre-dating time until the time point for onset of symptoms of RA, from pre-symptomatic individuals who donated multiple blood samples pre-dating the onset of symptoms of RA (*n* = 422) and from patients with RA (*n* = 192). Logarithmic mean concentrations during 2-year periods of anti-RgpB and anti-CPP3 antibodies in pre-symptomatic individuals, patients with RA and controls (*n* = 198) (**a**). Accumulated percentages of antibody positivity for anti-CPP3, anti-CCP2, anti-cfibrinogenβ36-52 (*cFibβ36-52*), anti-CEP-1 (α-enolase) and anti-cfilaggrin (*cFilaggrin*) antibodies in pre-symptomatic individuals and in patients with RA (**b**). *0* time point for onset of RA symptoms

Anti-RgpB IgG levels increased over time until symptom onset of RA, with a significant increase observed when analysing individuals with four consecutive pre-dating samples (*p* < 0.05; data not shown). There was a trend towards lower levels following diagnosis of RA (*p* < 0.088) (Fig. 2a). Similar to the anti-RgpB IgG response, the levels of anti-CPP3 IgG were found to increase constantly over the pre-dating time (Fig. 2a). However, no relationship was found between anti-CPP3 antibody positivity and the levels of anti-RgpB IgG (data not shown). The mean concentration of anti-RgpB antibodies in pre-symptomatic individuals (*n* = 64) exceeded that of the controls already 12 years before symptom onset, whilst the corresponding time for anti-CPP3 (*n* = 126) was 8 years before symptom onset. However, anti-CPP3 antibody concentrations above the mean value of the controls were detected in 11 out of 64 (17.2 %) pre-symptomatic individuals more than 10 years before symptom onset. A significantly higher number of pre-symptomatic individuals (26/64, i.e. 40.6 %) had a concentration of anti-RgpB antibodies above the mean value of controls at this time point (*p* < 0.001).

### The anti-CPP3 and anti-RgpB antibody response in relation to the ACPA response

**Fig. 3:**
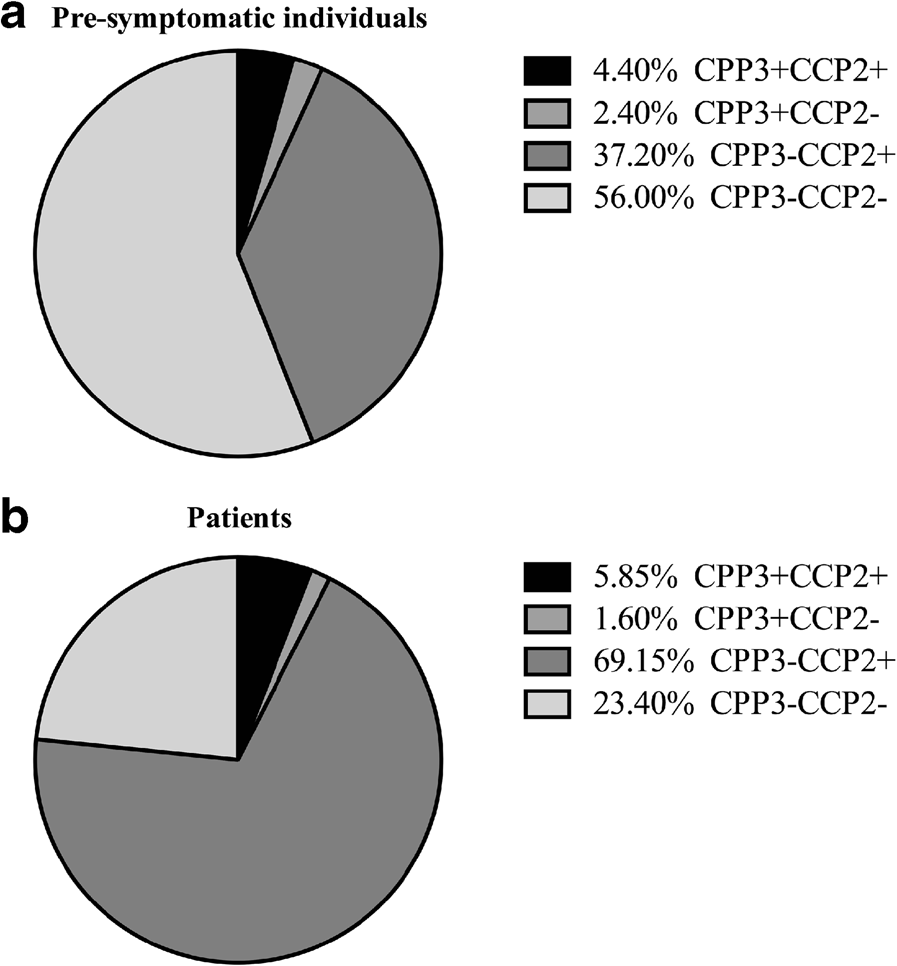
Pie charts illustrating the different sub-groups of pre-symptomatic individuals (one sample per individual, *n* = 251) (**a**) and RA patients (*n* = 188) (**b**), based on the presence/absence of anti-CCP2 and anti-CPP3 antibodies. Data presented as percentage

The accumulated frequency of anti-CPP3 antibody positivity increased constantly over time until symptom onset (Fig. 2b). This pattern mimics that of the “classical” ACPA response (defined as antibodies against CCP2, CEP-1, cFibβ36-52 and cfilaggrin) from the same time points, although at a lower frequency (Fig. 2b). The majority of anti-CPP3 IgG-positive RA patients (11 positive/15 analysed) and also pre-symptomatic individuals (11 positive/17 analysed) were confined to the anti-CCP2-positive subset (Fig. 3a, b, and Additional file 1: Table S1). In pre-symptomatic individuals, anti-CPP3 positivity was associated with positivity for anti-cFibβ36-52 antibodies (OR = 3.22; 95 % CI 1.24–8.36, *p* < 0.05), and anti-CPP3 antibody levels correlated with the concentrations of both anti-CCP2 (*r*_s_ = 0.14, *p* < 0.01) and anti-CEP-1 antibodies (*r*_s_ = 0.11, *p* < 0.05). The median pre-dating time for anti-CPP3 antibody positivity was closer to onset (−3.42 years) compared with anti-CCP2 (−4.56 years), anti-cFibβ36-52 (−5.17 years) and anti-CEP-1 (−3.49 years) antibody positivity. There was also a significant correlation between anti-RgpB IgG levels and anti-CEP-1 antibodies (*r*_s_ = 0.10, *p* < 0.05) in pre-symptomatic individuals (data not shown). No significant relationships were found between anti-RgpB or anti-CPP3 antibodies, respectively, and RF in the pre-symptomatic individuals or in RA patients (data not shown).

### Anti-RgpB and anti-CPP3 antibody responses in relation to cigarette smoking and RA risk genes

No associations were detected between anti-RgpB antibody levels and ever smoking in pre-symptomatic individuals, calculated in cases for whom several measurements were available or for the highest values of anti-RgpB antibodies (data not shown). In RA patients, both ever smoking and current smoking was associated with significantly lower levels of anti-RgpB antibodies (*p* < 0.012 and *p* < 0.019, respectively). No associations were identified between smoking and anti-CPP3 IgG positivity in pre-symptomatic individuals, or in RA patients (data not shown). Moreover, no associations were observed between carriage of *HLA-DRB1* SE or *PTPN22* T-variant and levels of anti-RgpB or anti-CPP3 IgG positivity, in pre-symptomatic individuals. In RA, *HLA-DRB1* SE was unrelated to the antibodies, while the *PTPN22* T-variant was associated with lower levels of anti-RgpB antibodies (*p* < 0.05; data not shown). However, caution should be taken when interpreting these data due to the low statistical power.

### Anti-RgpB and anti-CPP3 antibodies in relation to the development of RA

An association was identified between anti-RgpB antibody levels, stratified for above the 75th percentile vs below, in pre-symptomatic individuals (OR = 2.31; 95 % CI 1.41–3.78, *p* < 0.001) (Table 2). Analyses for ever smoking or carriage of *HLA-DRB1* SE or the *PTPN22* T-variant did not affect the OR (Table 2). Adjustments for age and sex in each of these analyses did not change the ORs (data not shown). Levels of anti-RgpB antibodies were not associated with having RA (OR = 1.20; 95 % CI 0.75–1.92, *p* = 0.44). Adjustments for smoking or *HLA-DRB1* SE or the *PTPN22* T-variant did not affect the association between anti-RgpB antibodies and RA (Table 2), and neither did further adjustments regarding sex and age (data not shown).

**Table 2:** Associations between anti-RgpB IgG (stratified for above the 75th percentile vs below) and the development of RA in pre-symptomatic individuals and RA patients

	Pre-symptomatic individuals (*n* = 251)				RA patients (*n* = 192)			
	Simple logistic regression	Multiple logistic regression			Simple logistic regression	Multiple logistic regression		
Variable	OR (95 % CI)	OR (95 % CI)			OR (95 % CI)	OR (95 % CI)		
Anti-RgpB	2.31 (1.41–3.78)	2.24 (1.35–3.71)	2.55 (1.67–4.43)	2.17 (1.28–3.68)	1.20 (0.75–1.92)	1.18 (0.73–1.92)	1.30 (0.77–2.17)	1.22 (0.74–2.00)
Ever smoker		1.88 (1.26–2.78)				2.14 (1.41–3.26)		
*HLA-DRB1* SE			3.58 (2.36–5.42)				3.14 (2.05–4.83)	
*PTPN22* 1858 T-variant				2.17 (1.36–3.48)				2.16 (1.33–3.53)

*OR* odds ratio, *CI* confidence interval, *RA* rheumatoid arthritis

Anti-CPP3 antibodies were not significantly associated with the development of RA in pre-symptomatic individuals, irrespective of analyses including smoking or *HLA-DRB1* SE or the *PTPN22* T-variant (data not shown) or with further adjustments for sex and age (data not shown). However, anti-CPP3 IgG was associated with RA, but only when adjusting for age, sex and *HLA-DRB1* SE (OR = 3.12; 95 % CI 1.06–9.19, *p* < 0.039) or the *PTPN22* T-variant (OR = 2.96; 95 % CI 1.02–8.57, *p* < 0.045) (data not shown). Adjustment for smoking, in addition to sex and age, was non-significant (OR = 2.66; 95 % CI 0.97–7.26, *p* = 0.056).

### Anti-CPP3 antibodies in combination with smoking or risk genes in the development of RA

When combining the major genetic risk factor for RA (*HLA-DRB1* SE) with anti-CPP3 IgG positivity, an increasing risk was observed for being pre-symptomatic (OR = 6.74; 95 % CI 1.43–31.81) compared with *HLA-DRB1* SE-positive/anti-CPP3-negative individuals (OR = 3.55; 95 % CI 2.32–5.42), although no significant interaction between the two factors was found (Table 3). Smoking in combination with anti-CPP3 antibody positivity showed no association with being pre-symptomatic (OR = 2.83; 95 % CI 0.86–9.4) (Table 3).

**Table 3:** Association of smoking or *HLA-DRB1* SE and anti-CPP3 IgG in pre-symptomatic individuals compared with controls adjusted for age and sex

	Anti-CPP3 IgG	Pre-symptomatic individuals, *n* (%)	Controls, *n* (%)	OR (95 % CI)
Smoking^a^				
–	–	84 (33.6)	90 (49.7)	Reference
+	–	149 (59.6)	85 (47.0)	2.12 (1.39–3.23)
–	+	6 (2.4)	2 (1.1)	3.01 (0.58–15.47)
+	+	11 (4.4)	4 (2.2)	2.83 (0.86–9.40)
*HLA-DRB1* SE^b^				
–	–	79 (31.6)	107 (62.6)	Reference
+	–	154 (61.6)	59 (34.5)	3.55 (2.32–5.42)
–	+	6 (2.4)	3 (1.8)	2.41 (0.58–10.13)
+	+	11 (4.4)	2 (1.2)	6.74 (1.43–31.81)
RERI = 2.21 (−8.13, 29.19), AP = 0.30 (−3.22, 0.55), SI = 1.52 (0.22–10.90), MI = 0.77 (0.10–6.29), *p* value = 0.81				

^a^Defined as ever smoker

^b^ *HLA-DRB1* shared epitope (*SE*) defined as 0101/0401/0404/0405/0408

*OR* odds ratio, *CI* confidence interval, *RERI* relative excess due to interaction, *AP* attributable proportion due to interaction, *SI* synergy index, *MI* multiplicative interaction

In RA patients, smoking combined with anti-CPP3 IgG increased the OR significantly from 1.73 (95 % CI 1.10–2.72) in anti-CPP3 IgG-negative ever smokers to 3.61 (95 % CI 1.05–12.44) in anti-CPP3 IgG-positive ever smokers (Table 4). *HLA-DRB1* SE also yielded significantly higher OR in combination with anti-CPP3 IgG positivity than in combination with anti-CPP3 negativity (OR = 8.80; 95 % CI 1.80–43.03 vs OR = 3.33; 95 % CI 2.11–5.23) (Table 4). However, no significant interactions were observed between smoking and anti-CPP3 IgG, or between SE and anti-CPP3 IgG. Carriage of the *PTPN22* T-variant in combination with anti-CPP3 antibody positivity revealed no significant association with the development of RA in patients or pre-symptomatic individuals (data not shown). Current smoking yielded similar results to ever smoking, although slightly weaker associations, which could be due to fewer current smokers compared with ever smokers, which also include former smokers.

**Table 4:** Association of smoking or *HLA-DRB1* SE and anti-CPP3 IgG in patients with RA compared with controls adjusted for age and sex

	Anti-CPP3 IgG	RA patients, *n* (%)	Controls, *n* (%)	OR (95 % CI)
Smoking^a^				
–	–	58 (30.2)	90 (49.7)	Ref.
+	–	119 (62.0)	85 (47.0)	1.73 (1.10–2.72)
–	+	5 (2.6)	2 (1.1)	4.04 (0.73–22.32)
+	+	10 (5.2)	4 (2.2)	3.61 (1.05–12.44)
RERI = −1.17 (−18.21, 8.19), AP = −0.30 (−7.38, 0.46), SI = 0.71 (0.08–6.55), MI = 0.46 (0.059–3.59), *p* value = 0.46				
*HLA-DRB1* SE^b^				
–	–	64 (33.3)	107 (62.6))	Ref.
+	–	113 (58.9)	59 (34.5)	3.33 (2.11–5.23)
–	+	6 (3.1)	3 (1.8)	3.55 (0.84–15.03)
+	+	9 (4.7)	2 (1.2)	8.80 (1.80–43.03)
RERI = 1.98 (−10.10, 30.25), AP = 0.26 (−3.96, 0.57), SI =1.44 (0.18–11.19), MI = 0.70 (0.08–5.81), *p* value = 0.74				

^a^Defined as ever smoker

^b^ *HLA-DRB1* shared epitope (*SE*) defined as 0101/0401/0404/0405/0408

*OR* odds ratio, *CI* confidence interval, *RERI* relative excess due to interaction, *AP* attributable proportion due to interaction, *SI* synergy index, *MI* multiplicative interaction

## Discussion

In the present study we investigated the role of the oral pathogen *P. gingivalis* in the development of RA, by focusing on the anti-*P. gingivalis* antibody response in RA patients prior to onset of symptoms. Our recent data show elevated antibody levels against the potent *P. gingivalis* virulence factor arginine gingipainB in patients with RA, especially in ACPA-positive RA [21]. We have also shown that these antibodies are clearly elevated in patients with PD, compared with periodontally healthy individuals, demonstrating that anti-RgpB IgG probably represents a good surrogate marker for *P. gingivalis* infection, which has been associated with PD [22, 23]. With the present study, we report increased concentrations of these antibodies in a subset of individuals years before onset of symptoms of RA. Consistent with Quirke et al.’s data in RA [25], concentrations of anti-CPP3 antibodies directed against, a synthetic cyclic citrullinated peptide  derived from another *P. gingivalis*-specific virulence factor, *P*.PAD, were also increased in a subset of both pre-symptomatic individuals and RA patients, compared with controls.

In line with our previous findings in RA patients, the association between the development of RA in pre-symptomatic individuals and the anti-RgpB antibody response was not dependent on smoking habits or presence of *HLA-DRB1* SE or the *PTPN22* T-variant [21]. Our data on lower anti-RgpB IgG levels in RA patients who were ever smokers or current smokers compared with non-smokers were also in line with a number of previous reports showing lower anti-*P. gingivalis* antibody levels in smokers compared with non-smokers [21, 39, 40]. One explanation for the observed trend of lower anti-RgpB antibody levels in RA patients compared with pre-symptomatic individuals (Figs. 1a and 2a) could therefore potentially be the higher frequency of smokers among RA patients (67.2 %), compared with pre-symptomatic individuals (64 %). Furthermore, as we recently showed for the anti-RgpB IgG response in RA, the *HLA-DRB1* SE in combination with anti-CPP3 IgG reveals a stronger association with RA and with being pre-symptomatic than *HLA-DRB1* SE alone. The same effect occurred when combining smoking with anti-CPP3 IgG positivity, although only in RA patients, not in pre-symptomatic individuals.

In this study, stratification of data into ACPA-positive and ACPA-negative sub-groups was not possible due to the limited number of individuals. However, a weak correlation between the concentration of anti-RgpB antibodies and that of the “classical” ACPA, measured as anti-CEP-1 antibodies, could be observed. Notably, ACPA (anti-CEP-1, anti-cFibβ36-52 and anti-cfilaggrin antibodies) were analysed by the ISAC multiplex assay, which is only a semi-quantitative method [37], and thus are not completely comparable with results from the ELISA used for measuring anti-RgpB IgG.

No relationship was detected between anti-RgpB and anti-CPP3 antibodies. This was unexpected considering the origin of both RgpB and CPP3 as *P. gingivalis*-specific antigens, and our interpretation of these two antibodies as surrogate markers for a *P. gingivalis* infection. Although the concentrations of anti-CPP3 antibodies, like anti-RgpB antibodies, were significantly increased in both RA patients and pre-symptomatic individuals compared with controls, the frequency of anti-CPP3 antibodies was only significantly increased in RA patients. Moreover, the anti-RgpB antibody response was elevated (compared with controls) several years earlier (12 years) than the anti-CPP3 antibody response (8 years). The anti-CPP3 antibody response was similar to the “classical” ACPA response; that is, there was no reactivity to the arginine-containing control peptide RPP3, the majority of anti-CPP3 antibody-positive cases were also anti-CCP2 antibody positive and the anti-CPP3 antibody response (both concentrations and the accumulated frequency of positive samples) increased gradually during the pre-dating time until symptom onset [37]. Taken together, these data may suggest that the anti-CPP3 antibody response, rather than being *P. gingivalis* specific, simply belongs to the generic ACPA response, or rather represents cross-reactivity with another citrullinated antigen. The low frequency of these antibodies (8 % in RA) could point to this. Moreover, in-vivo auto-citrullination by *P.*PAD has been debated [39], and the CPP3/RPP3 peptide—with its internal rather than C-terminal citrulline residue—may not represent an in-vivo-generated antigen. Still, the CPP3/RPP3 peptide sequence is bacterial derived and does not correspond to any human protein sequence.

Results recently published by Fisher et al. [41] conflict with ours in that they were unable to identify associations between anti-RgpB or anti-CPP3 IgG and pre RA. The two studies differ in several aspects that may explain the discrepant results: their study was based on a smaller study population (*n* = 103) than ours (*n* = 251, with 422 samples), with participants from a number of different southern European countries whilst our study subjects were recruited from a geographically defined area in northern Sweden; and not all of the pre-symptomatic individuals in Fisher et al.’s study were confirmed to develop RA, as they were in our study [41]. Additionally, the periodontal microbiota has been shown to vary between countries [42] and bacterial strain diversity has been described previously for *P. gingivalis* [43]. Altogether, these differences could contribute to the variability in the results of these studies.

Supporting our results, Mikuls et al. [44] reported increased concentrations of anti-*P. gingivalis* antibodies in high-risk individuals compared with controls. Also in accordance with our data is a study by de Smit et al. [45], in which elevated anti-*P. gingivalis* antibody levels were observed in arthralgia patients with RF or ACPA positivity compared with controls. However, anti-*P. gingivalis* antibody levels in de Smit et al.’s study were not higher in arthralgia individuals who developed RA compared with those who did not. This was not possible to evaluate in our study [45].

We believe this to be the largest population-based study analysing the anti-*P. gingivalis* antibody response in individuals before onset of symptoms of RA performed to date. Our study has limitations: the samples analysed were from different population surveys, and were not collected on a regular basis; no information regarding periodontal status or treatment was available, hence there was an inability to set a cut-off value for the anti-RgpB antibodies response; and it was not possible to investigate the relationship between PD and the development of RA. Also, data on the presence of *P. gingivalis* DNA were not available, and analysis of the anti-RgpB IgG and the anti-CPP3 IgG responses in relation to the presence of the bacteria was consequently not possible. However, as in our previous study [21], the anti-RgpB IgG response was interpreted as a surrogate marker for a *P. gingivalis* infection, past or present, whilst our data suggest that the anti-CPP3 antibody response, which follows the “classical” ACPA response, should be considered ACPA specific rather than a *P. gingivalis*-specific antibody.

## Conclusions

Our data demonstrate that antibodies against *P. gingivalis* are significantly increased in patients with RA compared with controls, and that these antibodies are detectable years before the onset of symptoms, supporting an aetiological role for *P. gingivalis* in the development of RA. Studies on larger cohorts with samples collected on a regular basis are needed for a deeper understanding of the relationship between *P. gingivalis*, anti-*P. gingivalis* antibodies, ACPA and the development of RA.

## Additional file

Additional file 1: Table S1. Presenting the frequency of ever positivity for anti-CPP3 antibodies in relation to positive/negative anti-CCP2 antibodies or ACPA. (DOCX 15 kb)

## Abbreviations

ACPA: Anti-citrullinated protein/peptide antibodies
anti-CCP: Antibodies against citrullinated peptide antibodies
CCP1: filaggrin307-324, citrullinated at position 13
anti-CEP-1: antibodies against α-enolase5-21 citrullinated at positions 9 and 15
anti-cFibβ36-52: antibodies against fibrinogenβ36-52 citrullinated at position 44
AU: Arbitrary units
CCP: cyclic citrullinated peptide
CI: Confidence interval
CPP3: Cyclic citrullinated peptide 3
ELISA: Enzyme-linked immunosorbent assay
HLA: Human leukocyte antigen
IQR: Interquartile range
OR: Odds ratio
PAD: Peptidylarginine deiminase enzyme
PD: Periodontitis
PTPN22: Protein tyrosine phosphatase non-receptor type 22
RA: Rheumatoid arthritis
RF: Rheumatoid factor
RgpB: Arginine gingipainB
ROC: Receiver operating characteristic
RPP3: Arginine-containing control peptide
SD: Standard deviation
SE: Shared epitope
SEM: Standard error of mean
