# Erratum to: Concentration of antibodies against *Porphyromonas gingivalis*is increased before the onset of symptoms of rheumatoid arthritis

**DOI:** 10.1186/s13075-016-1164-1

**Published:** 2016-11-04

**Authors:** Linda Johansson, Natalia Sherina, Nastya Kharlamova, Barbara Potempa, Barbro Larsson, Lena Israelsson, Jan Potempa, Solbritt Rantapää-Dahlqvist, Karin Lundberg

**Affiliations:** 1 Public Health and Clinical Medicine/Rheumatology, Umeå University, Umeå, Sweden; 2 Department of Medicine Solna, Rheumatology Unit, Karolinska Institutet, Stockholm, Sweden; 3 Department of Oral Immunology and Infectious Diseases, University of Louisville, School of Dentistry, Louisville, KY USA; 4 Department of Microbiology, and Malopolska Centre of Biotechnology, Faculty of Biochemistry, Biophysics and Biotechnology, Jagiellonian University, Krakow, Poland

## Erratum

Unfortunately, after publication of this article [1], it was noticed that the x-axis markers for Fig. 2a and b were incorrect. The corrected figure can be seen below.

**Fig. 2:**
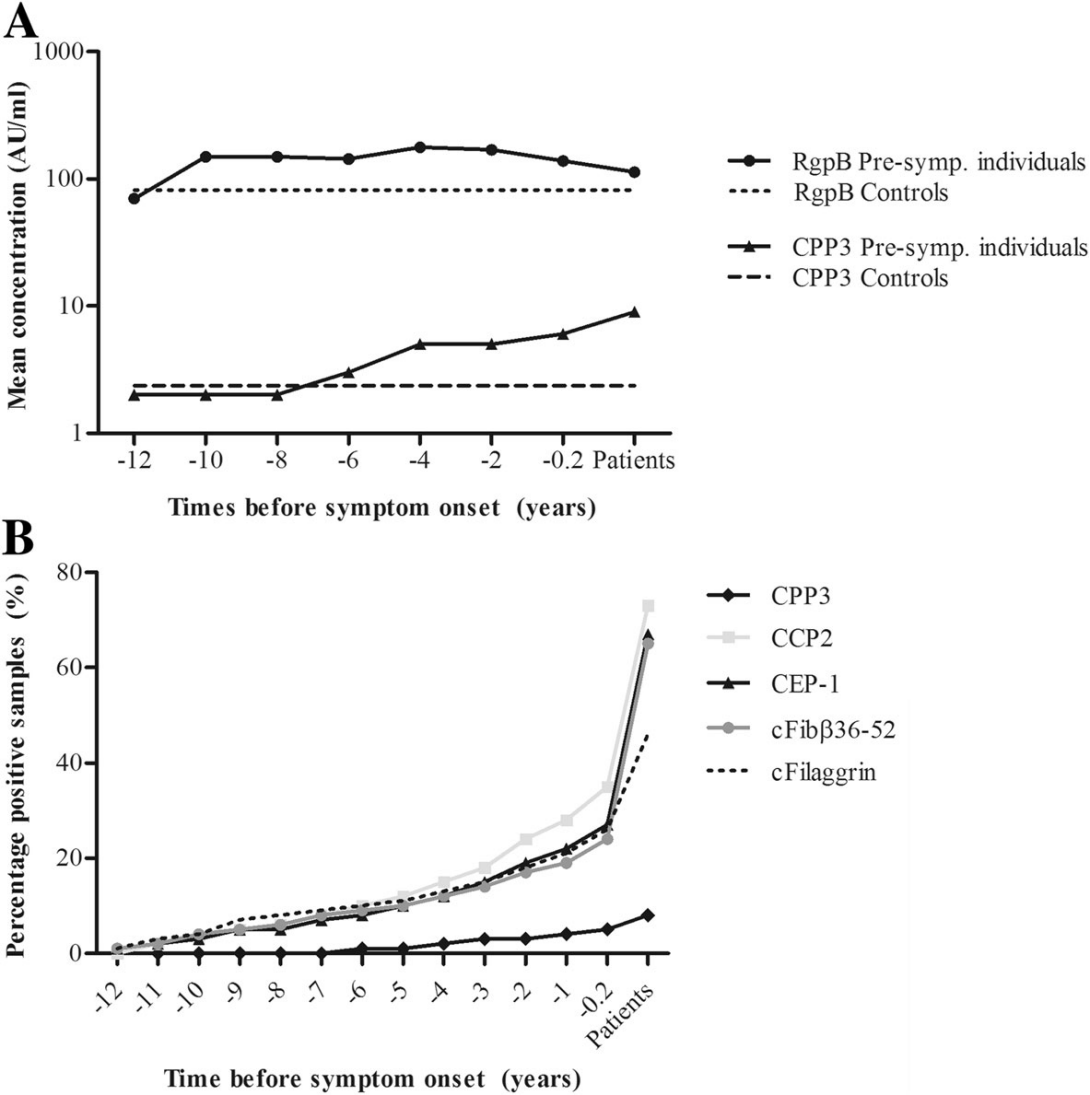
Antibody responses during the pre-dating time until the time point for onset of symptoms of RA, from pre-symptomatic individuals who donated multiple blood samples pre-dating the onset of symptoms of RA (*n* = 422) and from patients with RA (*n* = 192). Logarithmic mean concentrations during 2-year periods of anti-RgpB and anti-CPP3 antibodies in pre-symptomatic individuals, patients with RA and controls (*n* = 198) (**a**). Accumulated percentages of antibody positivity for anti-CPP3, anti-CCP2, anti-cfibrinogenβ36-52 (cFibβ36-52), anti-CEP-1 (α-enolase) and anti-cfilaggrin (cFilaggrin) antibodies in pre-symptomatic individuals and in patients with RA (**b**). 0 time point for onset of RA symptoms
