# Supplementary material for: Concentration of antibodies against Porphyromonas gingivalis is increased before the onset of symptoms of rheumatoid arthritis
Source: Arthritis Res Ther. 2016 Sep 7;18(1):201. doi: 10.1186/s13075-016-1100-4 (PMC5015325; doi:10.1186/s13075-016-1100-4)
Supplement: Additional file 1: Table S1. — Presenting the frequency of ever positivity for anti-CPP3 antibodies in relation to positive/negative anti-CCP2 antibodies or ACPA. (DOCX 15 kb) [file 13075_2016_1100_MOESM1_ESM.docx]

| **Additional file 1: Table S1. Frequency of ever positivity for anti-CPP3 antibodies in relation to positive/negative anti-CCP2 antibodies or ACPA** | | | | |
| --- | --- | --- | --- | --- |
|  | **Pre-symptomatic individuals, n=251** | | **RA patients, n=192** | |
| **Antibodies against** | **n (%)** | **Anti-CPP3+**  **n(%)** | **n(%)** | **Anti-CPP3+**  **n(%)** |
| **All individuals** | 251 (100) | 17 (6.8) | 192 (100) | 15 (7.8) |
| **CCP2+** | 105/251 (41.8) | 11/105 (10.5) | 141/188 (75.0) | 11/141 (7.8) |
| **CCP2-** | 146/251 (58.2) | 6/146 (4.1) | 47/188 (25.0) | 3/47 (6.4) |
| **CEP-1+** | 60/246 (24.4) | 7/60 (11.7) | 128/187 (68.4) | 10/128 (7.8) |
| **CEP-1-** | 186/246 (75.6) | 10/186 (5.4) | 59/187 (31.6) | 4/59 (6.8) |
| **cFibβ36-52+** | 83/247 (33.6) | 9/83 (10.8) | 124/187 (66.3) | 9/124 (7.3) |
| **cFibβ36-52-** | 164/247 (66.4) | 8/164 (4.9) | 64/187 (34.2) | 5/64 (7.8) |
| **CCP1+** | 78/245 (31.8) | 5/78 (6.4) | 88/187 (47.1) | 5/88 (5.7) |
| **CCP1-** | 168/245 (68.6) | 11/168 (6.5) | 99/187 (52.9) | 9/99 (9.1) |
